# Practice Effects on Repeated Self-Administered Digital Cognitive Assessments in Cognitively Intact Older Adults from the Framingham Heart Study: Longitudinal Observational Study

**DOI:** 10.2196/87208

**Published:** 2026-10-19

**Authors:** Kieffer Christianson, Chenglin Lyu, Cody Karjadi, Sherral Devine, Ting Fang Alvin Ang, Ileana De Anda-Duran, Vijaya B Kolachalama, Honghuang Lin, Huitong Ding, Katherine A Gifford, Rhoda Au, Phillip H Hwang, Preeti Sunderaraman

**Affiliations:** 1 Department of Psychology, University of Nevada, Reno, 1664 N. Virginia St, Reno, NV, 89557, United States, 1 (775) 784-6828; 2 Department of Anatomy and Neurobiology, Boston University Chobanian & Avedisian School of Medicine, Boston, MA, United States; 3 Framingham Heart Study, Boston University Chobanian & Avedisian School of Medicine, Boston, MA, United States; 4 Department of Neurology, Boston University Chobanian & Avedisian School of Medicine, Boston, MA, United States; 5 Department of Epidemiology, Tulane University School of Public Health and Tropical Medicine, New Orleans, LA, United States; 6 Department of Medicine, Boston University Chobanian & Avedisian School of Medicine, Boston, MA, United States; 7 Department of Computer Science, Boston University, Boston, MA, United States; 8 Department of Medicine, University of Massachusetts Chan Medical School, Worcester, MA, United States; 9 Department of Epidemiology, Boston University School of Public Health, Boston, MA, United States; 10 Boston University Alzheimer’s Disease Research Center, Boston University Chobanian & Avedisian School of Medicine, Boston, MA, United States; 11 Department of Psychiatry and Human Behavior, Memory and Aging Program, Butler Hospital, Brown University, Providence, RI, United States

**Keywords:** practice effects, brain smartphone, aging, digital assessment, cognitive assessment, neuropsychology

## Abstract

**Background:**

Remote, self-administered digital assessments provide scalable approaches for monitoring cognition in older adults. Practice effects (PEs), once viewed as measurement noise, are increasingly recognized as potential markers of cognitive decline. As disease-modifying therapies for neurodegenerative conditions emerge, longitudinal digital assessments may capture subtle cognitive changes. Although the majority of PE studies have focused on memory tasks, it is critical to determine whether PEs occur in other domains. Clarifying the potential presence of PEs across multiple time points on the Food and Drug Administration–cleared Defense Automated Neurocognitive Assessment (DANA) platform remains critical for determining whether PEs can serve as a measure of cognitive decline longitudinally.

**Objective:**

This study aimed to examine whether PEs occur and whether demographics moderate them across quarterly self-administrations of digital cognitive tasks spanning different cognitive domains in cognitively intact older adults.

**Methods:**

Cognitively intact older adults from the Framingham Heart Study self-administered DANA tasks at baseline and every 3 months for 1 year (4 assessments: T1-T4). Tasks included Simple Reaction Time (SRT), Go/No-Go (GNG), and Code Substitution (CS). These tasks varied in complexity, from simple motor responding (SRT) to inhibitory control (GNG) and associative learning with working memory demands (CS). The primary outcome was Cognitive Efficiency, a derived measure of speed and accuracy, which are also recorded as secondary measures. PEs were quantified using linear mixed-effects models, and the change in mean score across time points was expressed in pooled SD units as Cohen *d*.

**Results:**

A total of 717 participants completed all 4 time points (mean age 64.1, SD 9.5 years; n=469, 65.4% female participants; n=481, 67.1% college-educated). PEs were observed on all tasks, with small effects on CS (*d*=0.43) and GNG (*d*=0.32) and a below-threshold effect on SRT (*d*=0.16). Demographics influenced baseline performance: older age predicted lower baseline scores on all tasks, females scored lower on SRT and GNG, and higher education predicted better performance on all tasks. Age was the only demographic moderator of PEs, with smaller improvements on CS among participants aged 65 years or older (B=−0.39; *P*<.001).

**Conclusions:**

Quarterly self-administered digital cognitive assessments elicited measurable PEs across processing speed, inhibitory control, and visual scanning, with stronger effects on more complex tasks. Demographics influenced baseline performance, while age moderated PEs on the most demanding task. These findings provide baseline data against which PEs in clinical populations can be compared in future work, complementing prior evidence on other cognitive instruments.

## Introduction

Practice effects (PEs) refer to performance gains across repeated cognitive test administrations due to prior exposure to test materials and/or procedures [1]. Historically regarded as measurement noise, PEs are increasingly recognized as a meaningful index of learning capacity [2-4], and attenuated PEs have emerged as a candidate indicator of preclinical Alzheimer disease (AD) and prospective cognitive decline [5-8]. Failure to account for PEs can lead to misinterpretation of stable cognitive scores as preserved cognition rather than as reflecting deficits in learning capacity [9], and correcting for PEs alters rates of incident mild cognitive impairment detection [10].

PEs are mediated by test familiarity, whether with its stimuli or its procedures [2]. Stimulus-specific learning accumulates with each exposure [11], producing PEs that are largest on verbal memory tests and smallest on tests where new stimuli minimally alter task demands [12]. Alternate forms reduce but do not eliminate PEs on many tests [12,13]. The residual PEs reflect procedural familiarity with the test’s instructions and response format, which generalizes across stimuli. More complex tasks plausibly offer more procedural elements to learn, although task complexity has not been formally operationalized in PE research.

Moderators of PEs include the interval between sessions and individual characteristics. Recent digital research has used high-frequency, measurement-burst, and daily ecological momentary assessment designs that produce PEs within days to weeks [14-16], whereas earlier studies of legacy paper-and-pencil tests have documented PEs across intervals ranging from months to years [17-19]. Among individual characteristics, cognitive status is the strongest moderator of PEs [5,12,20,21]. Age also moderates PEs among healthy adults, with correlations between mean age and estimated PEs ranging from −0.66 to −0.89 across reasoning, spatial, memory, and speed domains [22]. Evidence on sex and education effects has been mixed in both traditional tests [23-25] and digital studies, with some reporting no demographic moderation [26] and others identifying task-specific associations [27-29].

The Framingham Heart Study (FHS) began collecting digital, self-administered, and remote serial cognitive data in 2020 using the Defense Automated Neurocognitive Assessment (DANA) application. Subsequent studies have demonstrated DANA’s feasibility and user acceptability [30,31], concurrent validity with traditional paper-and-pencil−based neuropsychological tests [32], and association with cardiovascular risk factors [33]. Although designed to minimize PE [34], Bellitti et al [27] reported modest response time (RT) PEs on 3 of 6 DANA tasks across 2 sessions spaced approximately 90 days apart in a 116-participant mixed-diagnostic sample. Whether these effects persist over additional administrations at a quarterly interval in a larger cognitively intact sample has not been established.

The primary aim of the current study was to characterize PEs across 4 quarterly assessments on 3 DANA tasks spanning a graded range of task demands (Simple Reaction Time [SRT], Go/No-Go [GNG], and Code Substitution [CS]) in a large sample of cognitively intact older adults. The secondary aim was to determine whether demographic characteristics moderate the observed PEs. The present study was not designed to test the prognostic value of PEs but rather to establish baseline PE patterns against which such questions can be evaluated in future longitudinal work. We anticipated that (1) PEs would be detectable across the 3 tasks but vary in magnitude, with more cognitively demanding tasks showing larger effects than motor-dominant tasks; and (2) older age would attenuate PE on the more demanding tasks [22], while sex and education effects would be modest given mixed prior evidence [23].

## Methods

### Participants

The study sample consisted of participants enrolled in the FHS, a longitudinal, population‐based cohort study that began in 1948 in Framingham, Massachusetts. The FHS focuses on studying risk factors for cardiovascular disease and other major chronic diseases spanning 3 generations of participants and their descendants. Neuropsychological evaluations began in 1976 with the collection of a brief cognitive screen and progressed to a comprehensive cognitive evaluation in 1999. The FHS began enrolling participants from diverse racial and ethnic backgrounds into multiethnic cohorts (Omni cohorts) in 1990, while the generation 2 (Gen 2) and generation 3 (Gen 3) cohorts are mostly White. This study included participants from 5 subcohorts of the FHS: Gen 2, Gen 3, Omni 1, Omni 2, and New Offspring Spouse. The inclusion criteria for this study were owning a smartphone, proficiency in spoken English, and having Wi-Fi access. All participants recruited for the study were presumed to be cognitively unimpaired based on established FHS dementia surveillance protocols, which flag participants for cognitive concerns based on Mini-Mental State Examination performance (score ≤24 or decline ≥3 points between visits), self-reported or informant-reported cognitive symptoms, or clinician referral, followed by consensus dementia review by a panel with at least one neurologist and one neuropsychologist [35]. Sociodemographic data were collected in accordance with established FHS protocols [32]. Variables included self-reported age (years) at the first assessment, sex (female/male), and education (high school or less, some college, and college graduate).

A total of 1756 participants completed at least one cognitive assessment during the data collection period (July 14, 2022, to March 28, 2025) and had the opportunity to complete all 4 quarterly assessments. The primary analytic sample comprised the 717 participants who completed all 4 time points of CS; corresponding completer counts were 701 for GNG and 686 for SRT, reflecting the fixed task order in which early discontinuation within a session resulted in retention of data for tasks completed earlier. The remaining 1039 participants completed 1 to 3 time points (partial completers) and served as the comparison sample for selection-bias analyses.

### DANA Tasks

Participants were asked to complete the DANA tasks every 3 months for a year, resulting in 4 time points (baseline plus 3 additional time points). DANA tasks were delivered via the Linus Health mobile app. For each assessment interval, participants had a 42-day window to complete their assessments. Participants received automated daily notifications if tasks remained incomplete, which they had the option to mute. Research assistants provided up to 2 to 3 additional reminders per assessment window via phone, email, or text, based on participant preference.

The DANA app is a Food and Drug Administration–cleared computerized neurocognitive test battery accessible as a mobile application on iOS and Android devices. Previous work has demonstrated DANA’s internal consistency and reliability [36]. Three subtests were selected for this study that collectively take approximately 5 minutes to complete. The first subtest, SRT, assesses RT by measuring latency between when a large target appears on the screen and when the participant taps the target across 40 trials. The second subtest, GNG, assesses sustained attention and impulsivity by requiring the participant to either tap or omit a response based on the color of stimuli that appear in varying locations on the screen across 30 trials. The third subtest, CS, assesses visual scanning, attention, and learning by asking the participant whether a sequence of symbol-digit pairs matches the 9 pairs shown in the key [34]. The same 9 symbol-digit pairs appeared at every administration; stimuli were not varied across the 4 time points. Task complexity is not a formally operationalized or quantified construct but rather reflects the polyfactorial demands imposed by a task, that is, the extent to which multiple cognitive domains are simultaneously engaged during task performance [27,37,38]. For reference, SRT requires only a simple motor response to a single stimulus and is therefore the least complex. GNG introduces additional inhibitory control and rule-based responding, while CS incorporates a learning component and subtle working memory demands, attributes recognized as markers of greater task complexity [37,38]. This progression is also reflected in average RT, with SRT producing the fastest responses, followed by GNG, and then CS [33].

The primary metric for DANA tasks is Cognitive Efficiency (CE), computed as percent correct divided by the mean RT for correct responses, multiplied by 60,000 to express the result in correct responses per minute units. This formulation derives from the throughput metric defined by Thorne [39], with DANA using correct-trial RTs only rather than the cumulative RTs across correct and incorrect trials in Thorne’s original metric. Most neuropsychological tests prioritize either speed or accuracy, leaving the other dimension less rigorously quantified. CE integrates both into a single performance index. CE was specified as the primary outcome for all PE analyses. Where CE-based analyses revealed a significant pattern, RT (milliseconds) and accuracy (percentage of correct responses) were examined as secondary outcomes to clarify which dimension was driving the observed effect.

### Analyses

Descriptive statistics are presented for the largest test sample (CS; n=717), which represents the union of all participants included in the analytic sample, with demographic characteristics summarized in Table 1. Descriptive statistics for demographic characteristics are presented as means and SDs for continuous variables and counts and percentages for categorical variables. Test descriptive statistics include means for RT, accuracy, and CE.

PEs were defined as improvements in task performance across repeated administrations and were operationalized in two ways. First, effect sizes (ie, Cohen *d*) were calculated to quantify the magnitude of observed change across time points in pooled SD units, providing a standardized comparison across tasks. Second, linear mixed-effects models (LMM) were used to test the significance of PEs and to examine potential demographic moderators. Thus, model coefficients (unstandardized B) represent the estimated average change in CE per time point, with CE specified as the primary outcome variable and time (4 assessments) as the primary predictor. For any observed demographic moderation, additional analyses were conducted examining RT and accuracy as secondary outcomes.

**Table 1.** Clinical characteristics (N=717).

Characteristic	Sample values
Age (years), mean (SD)	64.1 (9.5)
Education level, n (%)	
College graduate	481 (67.1)
Some college	172 (24.0)
High school or less	64 (8.9)
Sex, n (%)	
Female	469 (65.4)
Male	248 (34.6)
Race, n (%)	
White	669 (93.3)
Black	14 (2.0)
Asian	13 (1.8)
Asian Indian/Native Hawaiian/Pacific Islander	1 (0.1)
Other^a^	20 (2.8)
FHS^b^ cohort, n (%)	
Gen 3	515 (71.8)
Gen 2	131 (18.3)
Omni 2	41 (5.7)
Omni 1	20 (2.8)
NOS^c^	10 (1.4)

a Other includes Hispanic, more than one race, and prefer not to answer.

b FHS: Framingham Heart Study.

c NOS: New Offspring Spouse.

The main effect of time, representing PE while adjusting for age, sex, and education, was first tested using LMMs (*lme4::lmer*; R, version 4.3.1; R Foundation for Statistical Computing). Subsequent demographic models examined the influence of demographic variables at baseline and whether they moderated PE through interaction terms (time × demographic variable). To further evaluate age effects, an additional analysis dichotomized participants at 65 years of age for each DANA task. Age was modeled continuously in primary analyses; the dichotomized analysis used a conventional threshold of 65 years for older adulthood, yielding approximately balanced subgroups (55% aged 65 years or younger, 45% aged 65 years or older). Models included random intercepts for participants. Sensitivity analyses fitting random intercepts and slopes for time produced consistent fixed-effect estimates for the time term on CS and GNG, and an attenuated but directionally consistent estimate for SRT. Results from the random-intercept models are reported in the main text, and the results from the random intercepts and slopes are provided in Multimedia Appendix 1 (Tables S1 and S4).

To evaluate whether the analytic-sample restriction to participants who completed all 4 time points affected estimated PE slopes, the no-interaction main-effect-of-time model was refitted within subgroups of participants who completed 2 (n=302) or 3 (n=349) consecutive time points, separately for CS, GNG, and SRT. Results are reported in Multimedia Appendix 1 (Tables S1 and S3).

To formally test whether PE magnitude differed across tasks, a stacked LMM combining data from all 3 tasks was fit with task and time as fixed-effect predictors and a task × time interaction. Outcomes were standardized within each task by that task’s pooled SD across time points, such that interaction coefficients are interpretable in standardized effect size units comparable to the Cohen *d* values reported for individual tasks. Pairwise contrasts (SRT vs CS, SRT vs GNG, GNG vs CS) were derived from the interaction model (Tables S1 and S5 in Multimedia Appendix 1).

Baseline characteristics were compared between completers (n=717) and partial completers (n=1039) to evaluate selection bias. Demographic comparisons used unadjusted 2-group tests (Welch *t* test for continuous variables and chi-square tests for categorical variables). Baseline (T1) DANA cognitive scores (CE, RT, and accuracy) on each task were compared using linear models adjusted for age, sex, and education to isolate cognition-specific differences from those driven by demographic imbalance (Tables S1 and S2 in Multimedia Appendix 1).

### Ethical Considerations

The procedures and protocols of the FHS were approved by the institutional review board of the Boston University Medical Campus (H-40147). Written informed consent was obtained from all participants. The analyzed data did not include personal identifiers and were linked to a randomly generated identification number that cannot be used to identify participants personally. Study investigators never received or had access to identifying information. Since no identifying information was collected and no linkage file exists, there is no reasonable way to deduce participant identities from the data. The standard operating procedures used to protect the security of the data, along with other protections, are more than adequate to ensure the privacy of research participants and the confidentiality of the data. Participants were not financially compensated for participating in the study.

## Results

### Descriptive Characteristics

The sample comprising 717 participants (Figure 1; mean age 64.1, SD 9.5 years) was mostly White (n=669, 93.3%), female (n=469, 65.4%), and college graduates (n=481, 67.1%), primarily from the Gen 3 cohort (n=515, 71.8%, Table 1). Mean accuracy for T1 and T4 and the change in RT were as follows: for SRT, accuracy was 98.83% at T1 and 99.44% at T4, and RT decreased from 375.76 to 363.80 milliseconds; for GNG, accuracy was 96.81% at T1 and 97.36% at T4, and RT decreased from 883.46 to 843.95 milliseconds; and for CS, accuracy was 95.96% at T1 and 97.27% at T4, and RT decreased from 1941.46 to 1788.82 milliseconds (Table 2). Accuracy and RT showed an inverse relationship across tasks such that CS had the lowest accuracy and highest RT, SRT the highest accuracy and lowest RT, and GNG was intermediate on both. These descriptive trends corresponded to higher composite CE scores across assessments (Table 2).

Compared with partial completers (n=1039), the participants who completed all 4 time points were older (mean age 64.1, SD 9.5 years vs mean age 61.4, SD 10.1 years; *P*<.001), more often female (469/717, 65.4% vs 566/1039, 54.5%; *P*<.001), and more highly educated (481/717, 67.1% vs 101/1039, 9.7% college graduates; *P*<.001), with no difference in racial composition (*P*=.39; see Table S1 in Multimedia Appendix 1). After adjustment for age, sex, and education, groups did not differ in T1 CE, the primary outcome, on any task (see Tables S1 and S2 in Multimedia Appendix 1).

**Figure 1.**
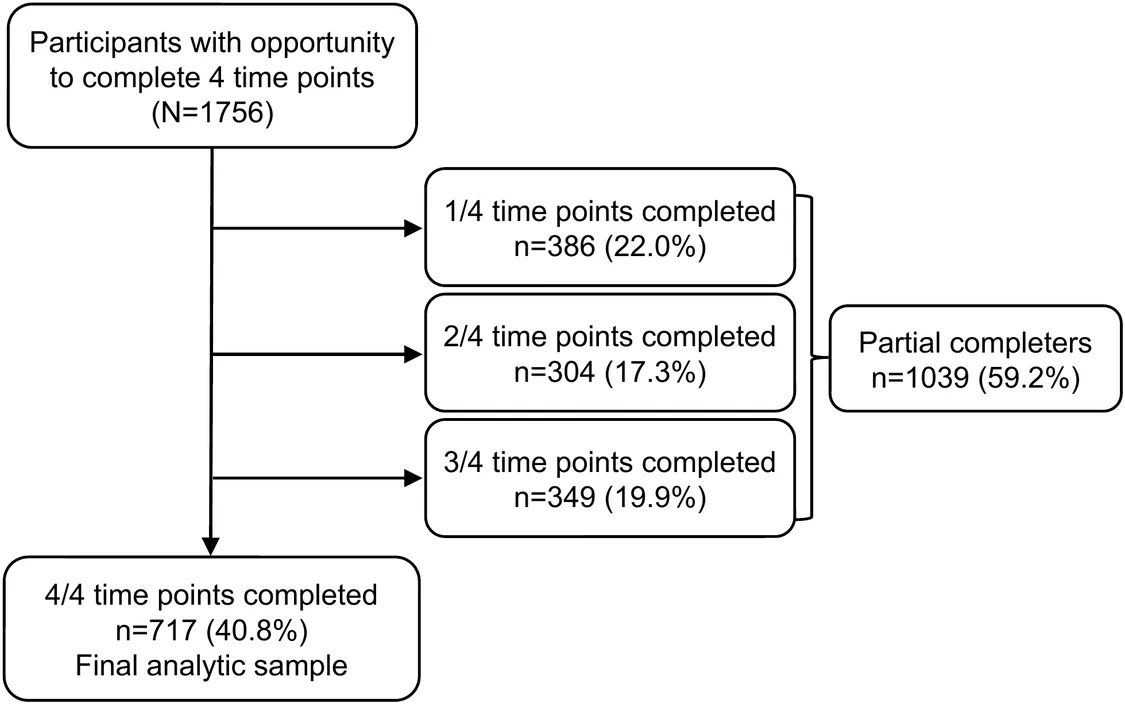
Participant selection based on adherence to the assessment schedule.

**Table 2.** DANA^a^ subtest descriptives.

Task and visit	RT^b^ (milliseconds), mean (SD)	Accuracy (%), mean (SD)	CE^c^, mean (SD)
SRT^d^			
T1	375.76 (78.01)	98.83 (6.27)	164.84 (30.23)
T2	366.78 (69.80)	99.37 (2.49)	168.09 (27.67)
T3	365.48 (68.44)	99.35 (4.31)	168.71 (27.31)
T4	363.80 (66.70)	99.44 (1.95)	169.43 (26.86)
Average^e^	367.95 (70.74)	99.25 (3.76)	167.77 (28.02)
GNG^f^			
T1	883.46 (134.08)	96.81 (12.02)	93.52 (22.91)
T2	865.96 (130.40)	96.86 (11.68)	96.42 (23.23)
T3	851.95 (118.77)	97.59 (9.51)	99.50 (22.19)
T4	843.95 (127.36)	97.36 (9.17)	100.68 (22.61)
Average^e^	861.33 (127.65)	97.16 (10.59)	97.53 (22.74)
CS^g^			
T1	1941.46 (404.04)	95.96 (9.08)	31.22 (6.91)
T2	1857.82 (388.85)	96.50 (6.90)	32.77 (7.27)
T3	1814.56 (393.21)	97.00 (5.52)	33.82 (7.50)
T4	1788.82 (382.85)	97.27 (3.84)	34.34 (7.58)
Average^e^	1850.67 (392.24)	96.68 (6.34)	33.04 (7.32)

a DANA: Defense Automated Neurocognitive Assessment.

b RT: response time.

c CE: Cognitive Efficiency.

d SRT: Simple Reaction Time.

e Average: mean of the means across the 4 time points; the value in parentheses is the mean of the SDs across the 4 time points.

f GNG: Go/No-Go.

g CS: Code Substitution.

### PEs

Without considering demographic influences, PEs on CE scores were observed across all subtests, reflected in significant main effects of time in the no-interaction models (Table 3). The magnitude of these PEs is shown in Figure 2, where improvements in CE from T1 to T4 were small for CS (*d*=0.43) and GNG (*d*=0.32) and below the small threshold for SRT (*d*=0.16). A stacked LMM with a task × time interaction confirmed that practice-effect magnitude differed across tasks, following the same ordering as the effect sizes, with all pairwise contrasts statistically significant (CS>GNG>SRT; see Tables S1 and S5 in Multimedia Appendix 1). Effect size trends in speed and accuracy indicated that changes for CS were driven by improvements in both RT and accuracy, while changes for GNG were driven almost exclusively by RT (Figure 2), reflecting baseline accuracy levels that were already at or near ceiling on GNG (mean 96.81%, SD 12.02%) and SRT (mean 98.83%, SD 6.27%). The slight decrease in cumulative effect size at T3 for SRT accuracy reflects a transient decline in performance between T2 and T3, though accuracy remained improved relative to T1 baseline. CS was the only subtest where change in accuracy reached the threshold for a small effect (*d*=0.21). For SRT, RT (*d*=0.17) and accuracy (*d*=0.16) contributed more evenly.

Sensitivity analyses, including those with partial completers (Tables S1 and S3 in Multimedia Appendix 1) and those fitting random intercepts and slopes for time (Tables S1 and S4 in Multimedia Appendix 1), showed no differences from the primary findings for CS or GNG; estimates for SRT were attenuated.

**Figure 2.**
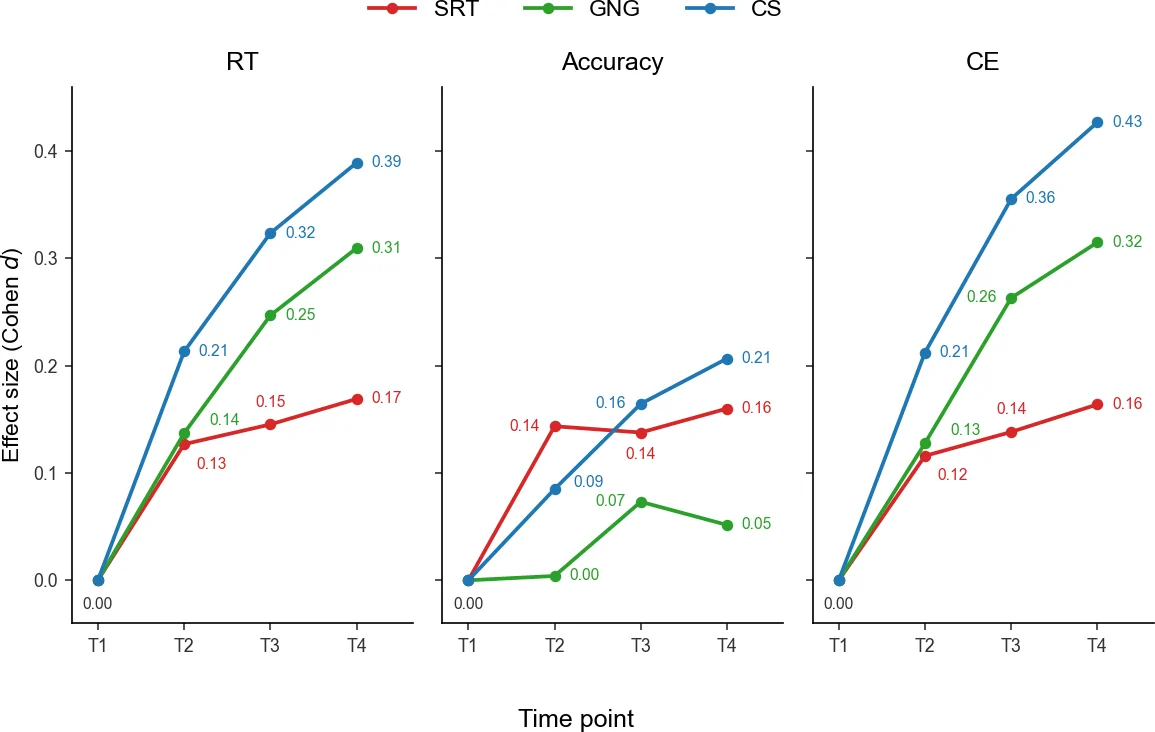
DANA subtest descriptives by effect size. Figures show the cumulative effect size. Cohen *d* thresholds: 0.20 (small effect), 0.50 (medium effect), and 0.80 (large effect). CE: Cognitive Efficiency; CS: Code Substitution; GNG: Go/No-Go; RT: response time; SRT: Simple Reaction Time.

**Table 3.** Practice effects models with demographic interactions.

Model and predictors	SRT^a^		GNG^b^			CS^c^	
	B^d^ (SE)	*P* value	B^d^ (SE)		*P* value	B^d^ (SE)	*P* value
No-interaction models							
Intercept	213.21 (6.92)	<.001	155.86 (4.77)		<.001	56.07 (1.57)	<.001
Time	1.64 (0.28)	<.001	2.73 (0.23)		<.001	1.15 (0.05)	<.001
Age models							
Intercept	212.41 (7.49)	<.001	152.59 (5.30)		<.001	53.53 (1.65)	<.001
Time	2.18 (1.93)	.26	4.91 (1.56)		.002	2.83 (0.34)	<.001
Age	−0.80 (0.10)	<.001	−1.03 (0.07)		<.001	−0.40 (0.02)	<.001
Time × age	−0.01 (0.03)	.78	−0.03 (0.02)		.16	−0.03 (0.01)	<.001
Sex models							
Intercept	214.24 (6.95)	<.001	156.08 (4.80)		<.001	56.08 (1.57)	<.001
Time	0.97 (0.49)	.045	2.58 (0.39)		<.001	1.15 (0.08)	<.001
Sex (female)	−5.80 (2.02)	.004	−3.64 (1.44)		.01	0.69 (0.44)	.10
Time × sex	1.02 (0.60)	.09	0.22 (0.48)		.65	0.00 (0.10)	.97
Education models							
Intercept	212.67 (7.07)	<.001	156.63 (4.91)		<.001	56.39 (1.59)	<.001
Time	2.02 (1.00)	.04	2.22 (0.79)		.005	0.93 (0.17)	<.001
Some college	3.26 (3.90)	.40	6.24 (2.75)		.02	2.31 (0.82)	.005
College graduate	9.85 (3.60)	.006	10.33 (2.52)		<.001	2.77 (0.75)	<.001
Time × some college	0.72 (1.15)	.53	0.43 (0.92)		.64	0.21 (0.20)	.29
Time × college graduate	−0.82 (1.06)	.44	0.61 (0.84)		.47	0.25 (0.18)	.16

a SRT: Simple Reaction Time.

b GNG: Go/No-Go.

c CS: Code Substitution.

d B: unstandardized fixed-effect estimate.

Age, sex, and education models showed varied patterns of demographic-related differences in baseline performance and in their influence on PE. Older age, modeled continuously, predicted lower baseline CE scores on all tasks. The only significant age × time interaction was on CS, indicating smaller PE with increasing age. To examine categorical age effects, participants were dichotomized into younger (≤65 years; 55% of the sample) and older (>65 years) groups (Table 4). This approach confirmed what was observed in the continuous models: age-related moderation of PE occurred only for CS (B=−0.39, SE=0.10; *P*<.001). Additional analyses examining differences in speed and accuracy demonstrated that this moderation was driven by accuracy (B=0.46, 95% CI 0.09‐0.83; *P*=.02) rather than RT (B=2.41, 95% CI −8.60 to 13.42; *P*=.67). Sex-related findings showed that at baseline, females scored lower than males on SRT and GNG, whereas no baseline sex-related differences were observed on CS. However, no significant sex × time interactions were observed, indicating that PE did not differ by sex. Education effects showed that, relative to participants with a high school degree or less, those with higher education scored better at baseline on all tasks. No significant education × time interactions were observed, indicating that PE did not vary by education.

**Table 4.** Difference in practice effects slopes for older participants (aged >65 years) compared with younger participants (aged ≤65 years).

Task	B^a^ (SE)	*P* value
SRT^b^	−0.93 (0.58)	.11
GNG^c^	−0.56 (0.47)	.24
CS^d^	−0.39 (0.10)	<.001

a B: unstandardized fixed-effect estimate.

b SRT: Simple Reaction Time.

c GNG: Go/No-Go.

d CS: Code Substitution.

## Discussion

### Principal Findings

PEs were observed across all 3 DANA tasks. The magnitude of PE increased with task complexity, likely due to the complex tasks provide greater opportunity for improvement. Among demographic variables, only age moderated performance over time, with older adults (aged 65 years or older) showing smaller PE on the CS task. This age-related moderation effect on the CS was driven primarily by accuracy rather than speed, which contrasted with the general trend of speed driving improvements in composite CE scores.

Early studies of DANA reported little evidence of PE [34,36,40], likely due to small samples, short retest intervals, preliminary practice sessions, and reliance on younger, predominantly male cohorts with high baseline performance. More recent work by Bellitti et al [27] found modest RT improvements across 2 sessions in older adults, with CS showing the largest effects and emerging as the only task differentiating cognitively intact from impaired participants. This pattern may reflect the polyfactorial nature of CS, that is, its simultaneous recruitment of multiple cognitive domains [37]. Our results are consistent with this pattern, with CS showing the largest PE, followed by GNG and then SRT in the current study. Direct comparison across studies is limited because Bellitti et al [27] did not report raw RTs and used a mixed sample of cognitively intact and impaired participants. Our larger sample revealed PE on both GNG and SRT, whereas the previous study observed no change on SRT. Prior studies have also found that CS performance relates to a broader set of abilities than SRT or GNG, with the CS task being associated with executive function, attention, processing speed, and memory [32]. Another study also found that the CS task was more strongly associated with a global measure of cognition, the Mini-Mental State Examination, than were SRT and GNG [41]. Interestingly, in a previous study, when the DANA app was administered remotely, the CS task could not be reliably completed by patients diagnosed with AD [42]. One reason could be that this task was particularly challenging to complete remotely without help for those with deteriorating cognitive abilities. Together, these findings support our description of CS as more complex than the other DANA tasks and suggest that this task might be a more sensitive task for detecting subtle impairment over time. Future longitudinal studies tracking these cohorts will be required to validate these suppositions.

Our findings also suggest that task complexity contributes to the presence of PE, but the unique contributions of speed and accuracy remain less clear. Bellitti et al [27] analyzed only speed, citing uniformly high accuracy at baseline as the reason for not examining accuracy. Our results confirm that most improvements across time were speed-driven but also indicate that the age-related moderation of PE on CS was more strongly linked to accuracy.

### Speed vs Accuracy

The ability of digital assessments to measure both speed and accuracy and to combine them into composite throughput measures [39] not only creates opportunities but also introduces new analytic challenges in interpreting higher-dimensional data. Cross-sectionally, variability in RT has been more closely linked to AD than accuracy [43], and tests with a majority speed-based component tend to produce the largest PEs [44]. However, Bergman et al [44] defined speed tasks using computerized iterations of legacy measures (ie, traditional neuropsychological tests) such as Trails A (approximately 20‐30 seconds), distinguishing them from simple reaction tasks such as SRT (approximately 3‐4 seconds), which, in the current study, is considered the most direct measure of speed. This distinction underscores the importance of carefully translating legacy constructs into digital formats. Relative to other measures in their respective eras, both Trails A and SRT are considered prototypical indicators of processing speed. Yet their susceptibility to PE differs, as Trails A is both more complex due to its demands on visual scanning and motor coordination and is substantially longer in duration. Bergman et al [44] also administered their tasks on laptops with external mice, whereas the present study relied on smartphones. Even within modern digital platforms, operating system and hardware differences (eg, iOS vs Android) can influence response capture and timing precision, raising additional concerns about generalizability across devices [45,46]. Thus, task duration, complexity, and device type likely increase susceptibility to PE, but disentangling their relative contributions is challenging. Whereas task duration is easy to quantify, task complexity is more abstract and difficult to measure directly. More broadly, this demonstrates that although tests are operationalized precisely, they often recruit multiple domains, and the relationship between tests and cognitive constructs is fluid [47].

### PE and Defining Change

This study adds to the growing literature on PE, including studies using digital cognitive assessments [20], traditional tests across short intervals [48], and those examining cognitive trajectories alongside concurrent biomarkers or lifestyle factors [49-51]. Much of the current high-frequency cognitive testing literature emphasizes memory tasks due to their sensitivity to neuropathological changes, particularly in AD [28,29]. In contrast, this study demonstrates that digital tasks measuring other predominantly nonmemory abilities, including processing speed, sustained attention, and visual scanning, also exhibit detectable PE.

Characterizing PEs in nonmemory tasks within a large sample of cognitively intact adults provides foundational data for research examining diminished PEs as a potential indicator of cognitive decline. Documenting that PEs occur in this population establishes a baseline for evaluating whether the absence or attenuation of PEs can distinguish impaired from intact individuals across neurodegenerative conditions and neurological injuries, where small but measurable deviations from normal trajectories may have clinical significance.

The utility of PE as a potential subtle indicator of cognitive impairment depends on careful consideration of assessment parameters, as PEs vary based on testing intervals, the specific cognitive domains assessed, the types of devices used, and how performance is quantified (speed, accuracy, or composite measures). Digital cognitive assessments vary considerably not only in the types of tests and cognitive abilities they measure but also in the time intervals between assessments, ranging from intensive longitudinal or measurement burst designs with multiple assessments in one day to weekly, monthly, quarterly, or annual follow-ups. Validation in cognitively impaired groups is a natural next step before PE from these brief digital tasks can be advanced as candidate clinical indicators.

The changes in performance observed in this study were significant but small in magnitude (SRT: *d*=0.16, GNG: *d*=0.32, CS: *d*=0.43). Given that effect sizes are expressed in SD units, these values reflect improvements of less than half an SD across 4 time points. Conventional neuropsychological interpretation relies on a ±1.645 SD threshold to define reliable change, a widely used but arbitrary cutoff intended to detect overt dysfunction [52]. Meanwhile, contemporary perspectives emphasize that neuropathological changes emerge before overt cognitive disruption, underscoring the potential relevance of smaller within-person shifts in cognition [7]. Progress toward this more nuanced perspective is reflected in recent efforts toward a “change-on-change” approach that interprets cognitive change alongside neuropathological change [49]. The current study clarified task-specific patterns of PE on DANA in a cognitively intact sample. Future research should aim to determine thresholds of clinical significance for PE, recognizing that meaningful change may be subtle and interpreted differently if occurring concurrently with neuropathological change.

### Strengths and Limitations

This study has several strengths. It represents the largest investigation of PE using the DANA platform, with participants completing 4 assessments across a 1-year period. This study contributes to the understanding of quarterly assessments, which is a relatively understudied temporal frequency situated between intensive longitudinal burst paradigms and long-interval observational studies. Another strength is the use of composite efficiency metrics that integrate both speed and accuracy, allowing both variables to contribute to the overall score.

There are also important limitations. The sample was predominantly White, female, and highly educated, which constrains generalizability. Analyses were conducted on summary scores generated by the DANA platform rather than item-level or trial-level data, which may have limited sensitivity to finer-grained practice-related changes. Also, to ensure consistency in examining PE, we only included participants who completed all 4 time points. Although sensitivity analyses of 2- and 3-time-point subsets (Tables S1 and S3, Multimedia Appendix 1) revealed statistically significant PE on CS and GNG among noncompleters, mirroring the ordinal pattern observed in the primary sample, the smaller effect magnitudes in these subsets suggest that estimates derived from the current completer sample may represent an upper bound on population-level PE among cognitively intact older adults. However, adherence to longitudinal testing is itself an important behavioral variable [53], and it remains unclear how missed or off-schedule assessments might influence the occurrence of PE. A comparison of baseline cognition between completers and partial completers showed no difference in CE after adjustment for age, sex, and education (Tables S1 and S2 in Multimedia Appendix 1). We also did not measure contextual factors such as comfort with the testing application [31] or momentary states such as arousal and distraction, which recent evidence indicates can affect cognitive performance in remote settings [54]. Finally, this study focused exclusively on cognitively intact participants without integrating biomarkers, limiting the ability to evaluate how PEs interact with underlying neuropathological changes. Future work could examine whether baseline cognitive performance moderates the magnitude of PE on these tasks.

### Conclusions

This study demonstrates that PEs are detectable across multiple digital cognitive tasks, with the largest effects observed on more complex tasks. Older adults showed smaller PEs on the CS task, and this trend was driven more by accuracy than speed. These results underscore the importance of considering task demands, as well as the relative contributions of speed and accuracy, when interpreting digital cognitive data. More broadly, the findings highlight the need to move beyond legacy thresholds for reliable change, which were designed to detect overt functional decline, and toward models that evaluate incremental within-person variability embedded within contextual factors as a meaningful signal in the aging process.

## Supplementary material

10.2196/87208 Multimedia Appendix 1 Analyses of sample characteristics, baseline cognitive scores, time effects, model specifications, and cross-task practice effects.

## Abbreviations

AD: Alzheimer disease
CE: Cognitive Efficiency
CS: Code Substitution
DANA: Defense Automated Neurocognitive Assessment
FHS: Framingham Heart Study
GNG: Go/No-Go
LMM: linear mixed-effects model
PE: practice effect
RT: response time
SRT: Simple Reaction Time
