# Supplementary material for: Practice Effects on Repeated Self-Administered Digital Cognitive Assessments in Cognitively Intact Older Adults from the Framingham Heart Study: Longitudinal Observational Study
Source: JMIR Aging. 2026 Oct 19;9:e87208. doi: 10.2196/87208 (PMC13679869; doi:10.2196/87208)
Supplement: Supplementary file 1 [file aging-v9-e87208-s001.docx]

**Multimedia Appendix 1**

**Table S1.**

*Demographic comparison between four-timepoint completers and partial completers*

| Characteristic | Completers  (*n* = 717) | | Partial completers (*n* = 1,039) | *P* |
| --- | --- | --- | --- | --- |
| Age, years, mean (SD) | 64.1 (9.5) | 61.4 (10.1) | | <.001 |
| Sex, *n* (%) |  |  | | <.001 |
| Female | 469 (65.4) | 566 (54.5) | |  |
| Male | 248 (34.6) | 473 (45.5) | |  |
| Education, *n* (%) |  |  | | <.001 |
| College graduate | 481 (67.1) | 101 (9.7) | |  |
| Some college | 172 (24.0) | 257 (24.8) | |  |
| High school or less | 64 (8.9) | 679 (65.5) | |  |
| Race, *n* (%) |  |  | | .39 |
| White | 669 (93.3) | 969 (93.3) | |  |
| Black | 14 (2.0) | 23 (2.2) | |  |
| Asian | 13 (1.8) | 10 (1.0) | |  |
| Asian Indian / Native Hawaiian / Pacific Islander | 1 (0.1) | 1 (0.1) | |  |
| Other | 20 (2.8) | 36 (3.5) | |  |

*Note. SD = standard deviation. Demographic comparisons used Welch's t test for continuous variables and chi-square tests for categorical variables.*

**Table S2.**

*Baseline (T1) DANA cognitive scores compared between four-timepoint completers and partial completers, adjusted for age, sex, and education*

| Task | Outcome | Completers,  mean (SD) | Partial completers, mean (SD) |  | *P* |
| --- | --- | --- | --- | --- | --- |
| CS | CE | 33.0 (7.4) | 33.0 (7.7) |  | 0.79 |
|  | RT | 1850.7 (396.4) | 1853.7 (407.3) |  | 0.77 |
|  | ACC | 96.7 (6.6) | 96.3 (7.3) |  | 0.04 |
| GNG | CE | 99.0 (23.2) | 99.7 (23.0) |  | 0.21 |
|  | RT | 851.8 (128.3) | 849.0 (127.7) |  | 0.34 |
|  | ACC | 97.0 (11.0) | 96.9 (11.5) |  | 0.81 |
| SRT | CE | 168.1 (28.3) | 168.6 (28.1) |  | 0.40 |
|  | RT | 367.3 (71.4) | 365.6 (70.2) |  | 0.31 |
|  | ACC | 99.2 (4.4) | 99.2 (3.9) |  | 0.53 |

*Note. CS = Code Substitution; GNG = Go/No-Go; SRT = Simple Reaction Time. CE = cognitive efficiency; RT = response time (milliseconds); ACC = accuracy (percent correct). SD = standard deviation.*

**Table S3.**

*Time fixed-effect estimates on broader-sample subsets, age model*

| Task | Sample | *n* | *B* | *SE* | *P* |
| --- | --- | --- | --- | --- | --- |
| CS | 2-timepoint subset (T1+T2) | 302 | 1.23 | 0.42 | .005 |
|  | 3-timepoint subset (T1+T2+T3) | 349 | 1.83 | 0.34 | <.001 |
|  | Primary sample | 717 | 2.83 | 0.34 | <.001 |
| GNG | 2-timepoint subset | 302 | 1.21 | 0.28 | <.001 |
|  | 3-timepoint subset | 349 | 1.18 | 0.34 | <.001 |
|  | Primary sample | 701 | 4.91 | 1.56 | .002 |
| SRT | 2-timepoint subset | 302 | −0.05 | 0.30 | .873 |
|  | 3-timepoint subset | 349 | 0.14 | 0.29 | .642 |
|  | Primary sample | 686 | 2.18 | 1.93 | .260 |

*Note. CS = Code Substitution; GNG = Go/No-Go; SRT = Simple Reaction Time. B = unstandardized fixed-effect estimate for the time term from the age model (covariates: age, sex, education, time × age). SE = standard error. The 2-timepoint subset includes participants who completed only the first two consecutive timepoints (T1 + T2). The 3-timepoint subset includes only T1 + T2 + T3. Primary-sample rows are reproduced from main-text Table 3 for comparison.*

**Table S4.**

*Time fixed-effect estimates from random-intercepts-only versus random-intercepts-and-slopes specifications, age model*

|  | Random intercepts only |  |  | Random intercepts + slopes |  |  |
| --- | --- | --- | --- | --- | --- | --- |
| Task | *B* | *SE* | *P* | *B* | *SE* | *P* |
| CS | 2.83 | 0.34 | <.001 | 2.83 | 0.36 | <.001 |
| GNG | 4.91 | 1.56 | .002 | 4.92 | 1.57 | .002 |
| SRT | 2.18 | 1.93 | .260 | 2.20 | 2.04 | .280 |

*Note. CS = Code Substitution; GNG = Go/No-Go; SRT = Simple Reaction Time. B = unstandardized fixed-effect estimate for the time term from the age model (covariates: age, sex, education, time × age). SE = standard error.*

**Table S5.**

*Pairwise cross-task contrasts on practice-effect slopes (task × time interaction terms)*

| Pairwise contrast | *β* | *SE* | *P* | Direction |
| --- | --- | --- | --- | --- |
| GNG vs CS | −0.03 | 0.01 | <0.02 | - |
| SRT vs CS | −0.09 | 0.02 | <0.001 | - |
| SRT vs GNG | −0.06 | 0.02 | <0.001 | - |

*Note. CS = Code Substitution; GNG = Go/No-Go; SRT = Simple Reaction Time. β = task × time interaction coefficient on within-task standardized outcomes (each task's outcome standardized using its own pooled SD across all four timepoints). SE = standard error. Negative β indicates a smaller practice-effect slope in the first-named task; the resulting ordering of practice-effect magnitude was CS > GNG > SRT.*
